# Being a safe place: a qualitative study exploring perceptions as to how a rural community hospice could respond to enactment of voluntary assisted dying legislation

**DOI:** 10.1186/s12904-021-00891-3

**Published:** 2022-01-04

**Authors:** Kirsten Auret, Terri Pikora, Kate Gersbach

**Affiliations:** 1 Rural Clinical School of Western Australia, University of Western Australia, M701 31 Stirling Terrace, Albany, Western Australia 6330 Australia; 2 Albany Community Hospice, Diprose Crescent, Albany, Western Australia 6330 Australia

**Keywords:** Death, Assisted, Death with Dignity, Respect, Hospice Care, Qualitative Research, Health Policy

## Abstract

**Background:**

There is a lack of research to guide the implementation of voluntary assisted dying legislation within a hospice setting. Furthermore, there is limited published information related to the expectations of the community and staff to assist decision making regarding voluntary assisted dying in a community hospice. The aim of this study was to explore the expectations of staff, volunteers and members of the community as how a rural Australian community hospice could respond in relation to imminent enactment of Voluntary Assisted Dying legislation.

**Methods:**

A total of 63 Hospice staff and volunteers and community members participated in 11 workshops. This qualitative study used the interpretive description method to analyse the workshop transcripts.

**Results:**

While there was not a consensus view on community expectation, there was agreement among the participants for respect for a patient’s individuality and choices. Furthermore, care offered in hospice needs to remain non-judgemental and patient focused regardless of whether voluntary assisted dying policy was implemented or not. Both opportunities and risks associated with implementation were identified by the participants.

**Conclusion:**

There was common ground around the respect for the dying person and the ideal of a “safe place” despite opposing views on what this may mean in practice. There is a need for clarity in organisational responses around policy, risk management, education, and staff support.

## Introduction

The Voluntary Assisted Dying (VAD) Act 2019 [1] was passed in Western Australia (WA) in 2019 with full enactment of the legislation scheduled for mid-2021. At that time, community approval for the new law was polled at 88% [2] and there was widespread interest in public consultations held by government prior to parliamentary debate with 867 participants in community forums and 541 written submissions [3]. The WA experience of legislative change has also been occurring in the wider national and international context with similar laws already passed in Canada (in 2016, referred to there as medical assistance in dying (MAiD) legislation) [4], the Netherlands, Belgium, Switzerland, Luxemburg, some states in the United States of America, New Zealand (2019), Spain (2021) [5] and other Australian states from 2018 to 2021. Many jurisdictions had a defined period for implementation prior to full enactment of their laws, allowing time for dialogue about how healthcare services will respond, however there is minimal literature on the interface between palliative care and legalised VAD to guide services in conversations with both internal and external stakeholders. Gerson’s recent systematic review states that the relationship between palliative care and VAD is not well understood and that there is a need for further research [6]. Recently published information on VAD implementation focuses on large centres in which palliative care provision is only one aspect. The experiences of a large comprehensive cancer centre in Seattle [7], a network of tertiary hospitals in Canada [8] and a tertiary public health service in Melbourne [9] reported internal debate, a focus on values, placing VAD within the context of end-of-life choices, and staff surveys as being important for their executive in coming to a policy position.

Community hospices are much smaller inpatient palliative care services, with only a few in Australia [10]. They are typically not-for-profit organisations with significant volunteer involvement and funding that is reliant on fundraising and philanthropy. With its 30-year history, the Albany Community Hospice (ACH) is the only remaining community-owned hospice operating in WA. It is a fully accredited, secular, rural, eight-bed, free-standing unit with specialist nursing care, managed by a volunteer Board. Medical care is provided by general practitioners with support from a visiting palliative care specialist. During 2020, 164 patients were admitted with a 9.4-day average length of stay, with 67% of admissions ending with death, and with case-mix-adjusted patient outcomes better than the national reference period [11, 12]. ACH relies heavily on the local community for practical and financial support [13]. Following passage of the Act, the Board was challenged to decide whether VAD occurring in ACH aligned with community expectations of a palliative care unit, however there was little data to guide their decision [14]. The aim of this study was to explore the expectations of staff, volunteers and community supporters as to how hospice could respond in relation to enactment of the legislation.

## Methods

This was a qualitative study which followed a naturalistic philosophy where it is accepted that there are socially constructed elements to human knowledge and the inquirer and the ‘object’ of inquiry will interact and influence one another. The interpretive description method was used to design the study and analyse information, as it values subjective and experiential knowledge and can work with realities that may be contradictory. It aims to identify a variety of possible approaches to a real world question and to bring these to as much consensus as possible to achieve a practical outcome [15, 16].

### Research team

The research team consisted of the authors of this paper and an experienced facilitator. KA is a palliative medicine academic physician who provides specialist consultation to general practitioners at ACH and is a medical advisory committee volunteer. She was a representative on the Ministerial Expert Panel which advised government on the original VAD bill [3]. TP has a long career in public health research, especially in community programmes and has no formal links to ACH. KG is the ACH research nurse. The facilitator (CG) is an independent, experienced consultant who worked with local government and community development and has previously delivered workshops to develop ACH’s strategic plan. An independent facilitator was felt necessary to allow participants, who may have known the researchers through personal or professional roles within a small town, to feel less influenced by these potential relationships. Team members examined their own beliefs, judgements and practices during coding and shared these reflections at all team meetings to consider if and how these may influence the research. All were agnostic to the Hospice’s final policy about VAD implementation.

### Sample

The three groups identified as potential key informants for their likely differing viewpoints and experiences around palliative care, hospice and VAD were ACH staff (inclusive of doctors), volunteers (inclusive of committee members) and community members who self - identified as a supporter or someone who had previously been touched by Hospice. The final participants recruited were a convenience sample from these targeted groups. Hospice staff and volunteers were recruited through posters, staff meetings and social media. Community members were recruited through social media, posters in local shopping centres, local newspapers and community radio.

### Data collection

The workshops were conducted by CG using a semi-structured discussion guide and were held in a university lecture room. A member of the research team was present to assist the facilitator and provide support to participants if needed but did not comment about the topics under discussion. The support available, which was never required, included helping participants to a quieter place if requested, offering to call a friend/family member, offering to contact the ACH support coordinator for a more detailed assessment of their distress or referral to a community counselling service or their own GP. Field notes were provided to the research team by the facilitator after all the workshops were completed.

The discussion guide was developed following literature review and feedback from an independent qualitative researcher. The workshops began with background information related to ACH, its mission and values, a summary of the Act and ground rules for the discussions. Six scenarios of how ACH could respond to a terminally ill inpatient who requested to die by VAD were then presented one after the other, randomly starting with a different scenario each workshop (see Appendix 1). The participants were encouraged to discuss their reactions, whether the scenario would meet community expectations and to offer alternative options. After two workshops the research team and facilitator met and decided that no changes to the guide were required.

The workshop discussions were voice-recorded then transcribed and de-identified by a professional transcribing service. Transcripts were not returned to workshop participants. Informed consent and a demographic questionnaire were completed by each participant before the workshops.

### Data analysis

Data were analysed using thematic analysis emphasising identification of codes from the workshop transcripts and then a collaborative process between researchers to create themes. The researchers began by independently reading and re-reading the workshop transcripts to derive initial codes. Team members then iteratively sorted and tagged the data into these codes by highlighting key words, sentences and/or paragraphs in Word. The coded transcripts were compared, and discrepancies discussed until consensus was reached. The codes were categorised to identify broad themes and further discussion was undertaken until agreement.

## Results

### Participants

There were 63 participants (2–11 per group) in 11 workshops (identified as W1 – W11 below) between July and September 2020. The mean duration was 1.7 h (range 1.6–2.4 h). Although grouping with peers was offered, no participant requested this. Ten groups were mixed staff, volunteers and community members, with one group consisting of staff only. No participant withdrew.

Descriptive information is shown in Table 1. The ages of attendees ranged between 23 and 87 years with a median of 61 years. While there was an even spread of years of association among community members, eight of the 11 nurses who participated had five or more years of association with ACH.

**Table 1:** Description of Workshop Attendees

	% (n)
Gender	
Female	80.9 (51)
Male	19.1 (12)
Relationship/role in ACH	
Community member	39.6 (25)
Nurse	17.4 (11)
Volunteer	15.8 (10)
Administration	6.3 (4)
Doctor	6.3 (4)
Hospice Committee member	3.1 (2)
Support Worker	3.1 (2)
Other	7.9 (5)
Length of Association with ACH	
> 5 years	36.5 (23)
1–5 years	34.9 (22)
< 1 year	15.8 (10)
No answer	12.6 (8)

### Themes

Participants did not have a consensus view on what the community would expect from Hospice in terms of VAD implementation. However, a number of themes emerged that could guide possible Board responses. Of note is that various views were usually framed in an overall context of respect for the patient’s individuality and choices, and that care would always need to remain non-judgemental and patient focused.

*The only thing that’s important really here, their views, their wants, their needs, and what does that say? What is most important to her? So, what is it she is looking for? What support does she need? (W1)*

#### Hospice must be a safe place

Hospice being a *“safe place”* (W1–11) was a recurring theme in all workshops as *“something the Hospice should protect”* (W5). A safe place was seen as one in which everyone could feel confident that they would not be exposed to discrimination, criticism, or any emotional or physical harm. However, there was no unified view as to what that concept of safety would mean for a VAD implementation decision, either for patients, staff, or volunteers.

#### Safety for patients and families

For some, ACH would remain safe for patients and families if VAD was not practised within the facility. Concerns were raised that patients could become increasingly fearful about admission to Hospice if VAD was adopted as *“people are already refusing palliative care because they don’t want to admit they are dying”* (W8) or hold beliefs that *“we’re going to be killing people up at the Hospice”* (W4). Concerns about cultural and religious safety were also raised.

*Hospice at the moment is seen as a safe place by cultural [groups], by regional community, by our religious communities. We feel that we could lose that very much - that feeling of safe place, and that’s how Aboriginals describe it when they come as well, and they’re happy to come to Hospice. It’s not hospital and it’s not their home. And will we lose that if we provide that space for self-administration. (W1).*

For others, policies referring a patient to somewhere outside Hospice for access to VAD was seen as unsafe for that patient through a perceived lack of support for their choice and negative judgements from staff: *“I think that a lot of people would question going to the Hospice, given that the choices that they may make wouldn’t be honoured”* (W7).

Irrespective of VAD occurring in Hospice or not, it was suggested that safety could be improved for patients and families. Timely, professional and consistent information should be available about *“all the options, support networks, and community resources”* (W4) and about local VAD policies. Patients and families could be supported by a *“dedicated VAD support coordinator”* (W2) from within staff whose role is to help people understand what was possible within the Hospice context, provide practical support and be an extra resource for resolving conflict. This conflict was seen as potentially occurring within a family but also between families who interact in shared spaces within the facility. It was felt important that patients were not left on their own to navigate *“through all the bureaucracy and telephoning people”* (W6) at the end of life. If VAD became part of ACH practice, support services need to be extended (e.g., peer support groups and specific bereavement support) and existing quality and safety processes be widened, with comments noting a complication or error is *“bound to happen in the real world”* (W6).

##### Staff and volunteer safety

Participants considered that a safe place for staff and volunteers, independent of specific VAD policy decisions, would require several foundations to be in place. These included everyone feeling secure that their views would be treated with *“mutual respect”* (W8) and there would be no judgement about individuals’ decisions and *“the rights for the staff to say no”* (W1). Clarity in policy and procedures was advocated, with *“no second guessing”* (W8) required, especially around conscientious objection to participation and staff responsibilities. Mechanisms for outside *“independent”* (W5) and *“anonymous”* (W8) counselling, such as an external Employee Assistance Programme, were suggested for safety. An ongoing education programme was also emphasized including sessions describing VAD legislation in detail and workshops to develop *“toolboxes and scripts”* (W5) to assist staff in responding appropriately and resolving issues as they arose. Other suggestions included clear processes for conflict resolution and moderating escalation; building teams that felt safe and could openly communicate; the provision of mentors; training in managing interpersonal distress; new processes in accreditation of doctors around scope of practice; and the creation of a dedicated team to lead change processes.

##### Organisational safety

This was also referenced as a consideration as to whether VAD could be seen as safe practice for a centre that aimed to be a “leader in the provision of expert palliative care” [17]. Some considered it would *“go hand in hand with that palliative care”* (W7), *“be the ultimate palliative care”* (W2) and *“that the community expectation was that this falls under palliative care”* (W2). For others, VAD was felt to be a practice that would expose ACH as *“being out of step… standing alone and doing the opposite to what the rest of the palliative care family are doing”* (W1) and contradicting *“the philosophy that Hospice in no way hastens death”* (W8).

#### Community expectations

While many recollected high public polling for VAD legislation, most reported that they *“actually don’t know”* (W8) whether the community expected that VAD could occur in ACH *“because community is not a homogenous mix”* (W10). Participants believed that there is *“so much unknown”* (W10) around VAD and considered this is a highly complex area, with the intersection of a patient’s experience of a terminal diagnosis, palliative care and VAD described as a *“minefield”* (W4), *“messy”* (W1) and *“often not black and white”* (W1).

However, it was felt that expectations would become clearer as practices became more established, and that as a smaller organisation ACH may have fewer constraints when developing policies related to VAD, hence should *“reflect better on what this community feels it needs. … And respond more quickly. … And appropriately”* (W6) and remain open to changing concerns.

With respect to specific positions, many participants agreed that the provision of basic VAD information only (e.g., WA navigator service contact number) would not meet community expectations, and would be seen as *“pretty much unfeeling, uncaring, minimally supportive of someone who’s in need”* (W4) especially if information provision was in a perfunctory manner:

*I think it’s a cop out really. The Hospice is there to give help at a time when it’s really needed. A lot of people probably know the members of the Hospice anyway - small country town - and to turn around and say, no, we don’t want to have anything to do with it. We will give you a phone number. I think that’s just a cop out. I really do. (W8).*

The scenario where self-administration of the lethal medication was supported within AHC but not practitioner administration, was also seen by many as an unacceptable compromise that would fail to meet any community expectations:

*This is sort of a halfway – half-in, half-out. I don’t think we felt it was a typically good way to go… You either offer the whole lot or don’t offer it, but that’s just – you know, bucket half empty. (W2).*

Some participants believed that the community would expect patients to have all their choices around VAD access supported within the facility, including in the assessment process, assistance in finding doctors with the suitable qualifications, and support for self-administration of oral medication or intravenous administration:

*Overwhelming the priority of Hospice should be to support patients’ wishes to access their rights under voluntary assisted dying legislation. (W4).*

All participants agreed that the community does expect to be able to receive holistic end-of-life care close to home, which for some, that would include an expectation of access to VAD from a local palliative care provider:

*A lot of our customers have come here to retire. This is the place where they want to finish off their life. If no one in town accepted this responsibility or went with this decision, that kind of impacts that whole family scenario as well. (W7).*

Concerns were raised where people would go instead if they chose VAD but the Hospice did not offer support around it. However, there was also an understanding that many people choose to return to their own home to die if possible.

Participants reported that provision of a VAD navigation service for those outside the local area would not be within expectations with concerns about costs, staffing and training, and a perception that the community may perceive this as unsafe involvement in VAD when the core business is providing palliative care.

#### Opportunities arising from VAD

Participants suggested that the VAD Act provided opportunities for ACH to play a leadership role in the community, including becoming an education provider about palliative care, *“putting additional resources into promoting palliative care”* (W2) and increasing awareness of the normal processes of dying.

It was also believed that the Hospice could *“take the initiative”* (W3) to lead discussions with other healthcare providers to support a local whole-of-system response for patients who wanted to access VAD. This was described as core business and a way to mitigate any risks around negative community perceptions:

*The community needed further education on what palliative care actually is and the difference between palliative care and voluntary assisted dying might not always be clear. (W3).*

Business development opportunities were seen in providing this education and in more support for patients at home. It was considered that the latter may include a fee-for-service model providing patients with more home-based palliative care than currently available and a nursing presence at the time the VAD substance is taken or administered such as a *“death doula”* (W2) role. It was suggested that *“we going to be a lot busier, a lot more demand”* (W9) although this was balanced with an understanding that *“history tends to show that more people get the go ahead for this than actually ever really do it”* (W4).

#### Risk to Hospice

It was seen that there may be a risk to Hospice’s bed occupancy if the Hospice offered VAD. Issues raised included the loss of being considered a safe place, a negative impact on the Hospice *“brand”* (W5), the *“stigma about going into the Hospice and not coming out”* (W7) and a perception that Hospice is promoting earlier deaths.

Negatives expressed by participants included that the possibility of ACH supporting VAD being described as *“a travesty”* (W5) with the *“potential to polarise the whole community”* (W3) and expose staff to *“abuse from any crackpot in the community”* (W9). The risk of degradation of the palliative care culture was also raised - *“there is a crossing and palliative care will never be the same again”* (W1).

Participants also mentioned the potential loss of fundraising revenue, support and goodwill during discussions about lack of uniform community views:

*It would be quite dangerous ground for a community group like ours, even with our fundraising, our community presence, that we’re going to be causing a bit of a division there. (W1).*

The possibility of a division between staff and volunteers was also discussed, and the chance of burnout if only a small number were willing to care for patients dying by VAD.

## Discussion

These workshops allowed participants with varying knowledge of palliative care to discuss scenarios and explore their expectations as to how a rural community hospice may prepare for enactment of VAD legislation. This is an important consideration in the rural context as inequity of access to VAD for non-metropolitan patients has been described internationally [18]. Like Blaschke et al., who reported differing perceptions of healthcare professionals as to where VAD sat in meeting their common goal of relief of suffering and facilitating a good death [19], our work also highlighted that a cross-community alliance can be formed around areas of shared commitment.

Participants were united in their compassionate reaction to the dying person and that care should be non-judgemental. However, while a common goal was agreed upon that prioritised Hospice remaining a “safe place”, there were opposing views on how to achieve this within the VAD context. For some, offering VAD in Hospice would create a haven for patient-centred care which prioritised individual choice, and a way of dying consistent with a person’s own needs, values and culture. Others discussed the risk that ACH would be increasingly perceived in the community as a place where dying is hastened, compounding myths that palliative care is only for those at the end-of-life and that treatments used for pain and distress shorten life [20].

Similar to our findings, the idea of safety as a theme has been raised in other studies, highlighting emotional burden, strained relationships, increased workload, fear and stigma [21]. Palliative care providers from Canada suggest that these concerns are valid, reporting significant challenges in providing palliative care after MAiD legalisation with moral ambiguity and provider distress, interprofessional team conflict and a negative impact on the perceived role of palliative care from the public and media [22]. Conversely, a recent paper from Victoria [23] argues that there may be “too much safety” with an over-emphasis on safeguarding coming at the expense of equity of access, which is already known to be less for those living rurally [18] and those of lower socioeconomic status [24].

Out of these differing conceptualizations of compassion and safety, came an understanding that there would not be a consensus opinion in the wider community as to whether VAD should occur within the Hospice. With this was a recognition of multiple risks to the organisation, including to its longer-term financial viability due to weakening community support, as there would be some who would not agree with whatever implementation decision was taken.

The focus on issues that are unknown is like that shared by Victorian colleagues ahead of VAD becoming legal in that state [21]. Their paper stressed “this is uncharted water for all of us” and discussed the challenges anticipated by the 1086 healthcare provider respondents. The findings around fear of increased conflict with colleagues, patients and families, higher workload and emotional burden resonate with our findings. Concerns around the unknown impact on organisational culture, the logistics of offering a service and the fallout of such a service on palliative care were again similar.

Our participants gave advice on areas they agreed upon, mainly in relation to Hospice’s role in community education and leadership around palliative care and dying. They advised patients and families should receive transparent policy information around what could be offered within the facility and be cared for by staff who were trained in these policies. Staff should also be non-judgemental and compassionate in communication around VAD questions, within an organisational culture that was supportive and proactive in managing risk and conflict.

### Strengths and limitations

Our research demonstrated that it is possible to safely ask stakeholders about this sensitive topic with facilitated workshops allowing participants to respond to each other and the content in a manner that was respectful of differences. A particular strength of this work is in the interaction between the community participants, staff and volunteers. There are no other published studies exploring VAD implementation in an inpatient facility that focus on community opinions.

The workshops were conducted among English-speaking people residing in an Australian rural town. If this research had been conducted outside this context, with people inclusive of other diversity, the resulting discussions may have raised different issues. Most of the groups had participants with both a general knowledge of palliative care and the legislation and those with a much deeper lived experience and expertise. This allowed a sharing of experiences within the groups that facilitated openness in the discussions.

## Conclusion

This paper summarises expectations from a range of stakeholders as to how a community Hospice Board may approach decision-making around VAD implementation. There were common alliances around respect for the dying person and providing a “safe place” despite opposing views on what this may mean in practice. The need for clarity in organisational responses around policy, risk management, education, and staff support were highlighted. It is envisioned this work may be useful for similar organisations to guide their decision-making and preparedness.

## Data Availability

Not applicable.

## Appendix

### Workshop Scenarios

It may help you to imagine a person for whom this could be relevant. Imagine Jacky: she is a 78-year-old farmer who has lived in Albany for the last 30 years and is now in Hospice for care due to pain from end stage cancer. She is interested in dying by voluntary assisted dying. She may have gone through the process of assessment at home already with her doctors, or she may ask her nurse about it when she is admitted. Let’s presume she will meet all the eligibility criteria.

Scenario 1: Hospice does not support voluntary assisted dying, but will provide information as required by the law: Jacky is told by her nurse and doctor that they cannot provide any support with voluntary assisted dying as Hospice has a position statement saying as much. They talk with her in a gentle way and continue to offer her the best palliative care they can. Jacky is provided with the telephone number of the Health Department’s voluntary assisted dying “navigator service” as it is required to do so as part of the law. Jacky and her family contact the service and she returns home to follow up the rest of the process at home, based on their advice.

Scenario 2: Hospice directly assists voluntary assisted dying, limited to self-administration: Jacky’s own GP is in charge of her care in Hospice. That GP is willing and able to assist her with all the assessment requirements. It will mean some paperwork, some conversations with Jacky’s family and arranging another doctor to visit who can provide the second opinion. All of this would usually take 9 days. The GP receives approval for Jacky from the Health Department and is able to prescribe the medication that Jacky will use to die by voluntary assisted dying. She has decided she will take it when she is ready. A few days later she takes the medication while in Hospice and dies. Her GP returns to write her death certificate and do more paperwork. The funeral director comes to collect Jacky’s body from Hospice and her family plan her funeral.

Scenario 3: Hospice directly assists voluntary assisted dying by supporting either GP administration or self-administration: Jacky and her GP follow the same path however she has requested that her GP give her the medication in Hospice by intravenous injection on a day of her choosing. The GP gets authority for this to occur and in the presence of the required witness, gives Jacky the medication and she dies. Her GP writes her death certificate and does more paperwork. The funeral director comes to collect Jacky’s body from Hospice and her family plan her funeral.

Scenario 4: Hospice directly assists voluntary assisted dying, but some staff and volunteers will not be involved. Hospice will support voluntary assisted dying through organising care that supports the decisions of staff and volunteers: Jacky’s GP is working with her to complete the voluntary assisted dying assessments and she plans to stay in Hospice for care. Jacky’s nurse and some of the volunteers says that they cannot give any support with voluntary assisted dying. The Hospice management arrange for a care plan and care team that considers both Jacky’s needs and the preferences of staff and volunteers. All the required assessments are completed and Jacky has access to voluntary assisted dying medication – perhaps oral or perhaps intravenous.

Scenario 5: Hospice directly assists voluntary assisted dying, but Jacky’s GP will not be involved. Hospice will support voluntary assisted dying through liaising with another GP: Jacky’s GP says that he cannot help with her requests for support with voluntary assisted dying. She is getting good palliative care in Hospice and feels much more comfortable, so doesn’t want to go anywhere else. She asks if the Hospice can help her find a GP that can be her coordinating doctor. The Hospice staff know which of their doctors would have the required training and qualifications and asks another doctor to come and see Jacky. The new doctor and a colleague who gives the second opinion do all the required assessments and Jacky has access to voluntary assisted dying medication – perhaps oral or perhaps intravenous.

Scenario 6: Hospice directly assists voluntary assisted dying in all ways that are requested by the patient and also acts as a navigator service: The Hospice decides that for people like Jacky, they have the skills to be the local “navigator service” for any patients who would like to find out more about the requirements of the legislation and how they can be assessed and supported. Hospice sets up a telephone service for anyone in the community to call.

## Abbreviations

ACH: Albany Community Hospice
GP: General Practitioner
MAiD: Medical Assistance in Dying
VAD: Voluntary Assisted Dying
WA: Western Australia
